# Outcomes of first versus third kidney transplantations: propensity score matching and paired subgroup analysis—a single-centre experience

**DOI:** 10.1007/s00423-020-02063-y

**Published:** 2021-01-17

**Authors:** Gábor Telkes, László Piros, József Szabó, Gergely Huszty, Katalin Eitler, László Kóbori

**Affiliations:** Department of Transplantation and Surgery, Semmelweis University, VIII. Baross u. 23, Budapest, H-1082 Hungary

**Keywords:** Kidney transplantation, Patient survival, Graft survival, Graft loss, Retransplantation, Survival, Mortality, propensity score matching analysis

## Abstract

**Background:**

In the Eurotransplant, 12.6% of kidney transplantations are a repeat procedure. Third transplants are significantly more complex than first and second ones. We compared the results of first (PRT) versus third (TRT) transplantations.

**Methods:**

Between 2011 and 2016, we performed 779 deceased donor adult kidney transplantations, 14.2% out of them were second, 2.6% (20) third, and 0.3% fourth. We compared the pre-, intra-, and postoperative data, kidney function, and survival rate.

**Results:**

Recipients of TRT were younger (53.4 vs. 47.3 *p* = 0.02). HCV infection rate (20%, *p* = 0.00) is ten times higher. The operation time is longer (132 vs. 152 min, *p* = 0.02), and delayed graft function is much more frequent (22.4% vs. 60%, *p* = 0.00). Induction therapy was given to every TRT (7.9% vs.100%), but as a result, the rejection rate was the same (~ 15%). Hospital stay is a week longer. Patient’s survival at 1, 3, and 5 years for PRT is 96.4%, 93.9%, and 91.2% and for TRT is 90%, 85%, and 78.4%, respectively (*p* = 0.023). TRT’s odds ratio of fatal outcome is 4.35 (1.5–12.5). Graft survival at 1, 3, and 5 years for PRT is 93.1%, 91.4%, and 90.3% and for TRT is 75%, 75%, and 75%, respectively (*p* = 0.020). TRT’s odds ratio of graft loss is 3.14 (1.1–8.9). Of PRT 85.76%, out of PRT 85.76%, while out of TRT 60% live with a functioning graft, p=0.00149.

**Conclusion:**

In a third transplant, both graft and patient survival are significantly inferior to primer ones. Careful selection is required to minimize the patient risk and graft loss.

## Introduction

Despite significant improvements in the last five decades, the real half-life of kidney grafts is still around 8 years, substantially shorter than projected half-lives [1]. Chronic graft failure is still a major problem, and especially younger recipients of the primer graft might need a second or third transplantation. The rate of repeat transplantation was increasing in the USA from 1996 to 2005, reaching 12.4%, while potential retransplant recipients represented 16.1% of all kidney candidates [2, 3].

In the Eurotransplant, 17.9% of those on the waiting list are listed for repeat kidney transplantation, and 13.7% of the procedures performed was a repeat one in 2019 (http://statistics.eurotransplant.org).

While mortality after a failing graft is considered to be high, the first retransplantation is associated with significantly reduced mortality rates [4–6].

Moreover, in several papers, the outcome of a second graft has been reported to be similar to the first one [7–9]. Others observed that the graft survivals for repeat deceased donor transplants were all significantly lower; the relative risk of graft loss was 1.18–1.24 [2].

Recipients of a third graft constitute a unique population among kidney patients. Patients are often highly sensitized, have limited surgical options, suffer from comorbidities e.g. atherosclerosis, virologic infections, and all other consequences of previous operations, immunosuppression, and long-lasting dialysis. These patients accumulate several risk factors associated with poor patient and graft outcome [10].

The surgery of these patients is always challenging. Several surgical approaches exist but there is no standard technique [11].

Reported results of third transplantations are a bit inconsistent. Some studies demonstrated a similar survival rate to that of primary transplants, at least for patient survival [10, 12, 13]. However, the majority of the literature agrees on inferior graft survival with a higher complication rate [11, 14–16].

The kidney transplantation program started in 1973 in Hungary, and today there is a constant need for repeat transplantations [17]. Accurate knowledge of prognosis may help in the judicious and responsible use of deceased donor kidneys.

## Aims

The aim of our study was to analyse our results of third transplantations and compare them with primary ones.

## Materials and methods

This is a single-centre, retrospective, observational study from the largest Hungarian kidney transplant centre within the Eurotransplant community, with an institutional experience of more than 5000 kidney transplants since 1973.

### Patients

Between 2011 and 2016, 779 adult, kidney alone, brain-dead deceased donor transplantations were performed and included. Out of them, 82.9% (646) were first, 14.2% (111) second, 2.6% (20) third, and 0.3% (2) fourth transplantations.

Nine TRT recipients shared the donor with a first or second recipient. We compared the outcomes of these nine pairs as “selected third” (sTRT) vs. “non-third” (nTRT), as a mixed group of 7 first and 2 second recipients. We have no data about the kidney pair of the other eleven third recipients.

We prospectively registered the pre-, intra-, and postoperative data:

- Sex, age, BMI, ECD, virology
- PRA, HLA mismatch, immunosuppressive therapy, rejection
- Surgical details and complications, hospital stay
- DGF, kidney function, graft, and patient survival. Graft survival was recorded from transplantation to graft failure: graftectomy or return to dialysis. Death censored graft survival was counted.

The end of the observation period is March 2019.

### Immunosuppression

Immunosuppression was given as per protocol. Maintenance therapy generally consists of tacrolimus, mycophenolic acid, and prednisolone triple therapy.

Induction therapy was given to all third and 7.9% of primer recipients, considering previous immunization, *p* = 0.000.

### Surgical considerations

According to our centre policy, we require preoperative pelvic angio-CT to visualize the vascular anatomy of the potential TRT recipient. At least one of the previous grafts has to be removed prior to waitlisting. Removal of the specific kidney graft depends on clinical circumstances. Our centre’s preferred site is the right iliac fossa for primer, and if possible, for third transplant as well. Third transplantations were performed by experienced senior transplant surgeons.

### Statistics

For descriptive statistical analysis, mean and median values, standard deviations, and absolute and relative frequencies were calculated. Qualitative data were compared by the Pearson Chi-square test. Quantitative variables were compared using Mann-Whitney *U* or Kruskal-Wallis test. Survival was analysed by the Kaplan-Meier method and compared with log-rank test. A *p* value of less than 0.05 was considered to be significant. Propensity score matching analysis was performed using logistic regression analysis, and then thirds were matched to primers with a 1:1 matching in propensity scores without replacement. The match tolerance was set to 0.1. Statistics were calculated by TIBCO Software Inc. (2018), Statistica (data analysis software system), version 13, and by IBM SPSS version 25.

## Results

### Donors

The demographic data of donors are presented in Table 1. TRT donors were younger (47.4 vs.52); there was no difference in BMI, CMV infection rate, or sex. The CMV infection rate represents Hungarian population data, amounting to 86% [18].

**Table 1:** Demographics of donors

Donors	PRT (646)	TRT (20)	*p*
Age, y (SD)	52.0 (11.7)	47.4 (9.8)	n.s. 0.073
female, %	44.7	40.0	n.s.
BMI	26.2	25.7	n.s.
CMV IgG pos %	82.8	80.0	n.s.
ECD, %	34	0	*0.01*

None of our third recipients received an ECD graft, while this rate is about 34% for primer grafts.

### Recipients

Recipients’ data are presented in Table 2. TRT recipients are significantly younger than PRT recipients, (47.3 years vs. 53.4 years). Third recipients got significantly more HCV (20% vs. 2.1%), and slightly more CMV infection. The PRA level was much higher (34.4% vs. 2.5%) in the TRT group.

**Table 2:** Demographics, virology, and immunology of the recipients

	All recipients			Paired recipients		
	PRT (646)	TRT (20)	*p*	nTRT (9)	sTRT (9)	*p*
Age, y (SD)	53.4 (12.6)	47.3 (9.8)	*0.016*	53.7	48.9	n.s. (0.27)
Female, %	40.1	30,0	n.s.	66.7	22.2	n.s.
BMI	26.4	25.0	*0.045*	27.8	26.0	n.s.
HCV pos, %	2.1	20.0	*0.000*	0	11.1	n.s.
CMV IgG pos, %	81.5	95.0	n.s.	62.5	100	n.s.
CMV mismatch donor pos. Recipient neg.	14.2% (92)	5% (1)	n.s.	22.2 (2)	0	*0.023*
Antigen mismatch	3.0	3.1	n.s**.**	2.4	2.8	n.s.
PRA level, % mean/median	2.5/0.0	34.4/44.0	*0.0000*	4.7/0	30.0/7.0	*0.0042*

Considered significant if *p* < 0.05

### Surgery, postop course, rejection

Details and exact numbers are presented in Table 3. The cold ischaemic time is the same (~ 14–15 h), or even shorter in the TRT group. The whole operative time is significantly longer in TRT (132 vs. 152 min) (verified by propensity matching analysis as well), with a significantly shorter handling time (42 vs. 35 min).

**Table 3:** Surgical details and postop. course

	All recipients			Paired recipients		
	PRT	TRT	*p*	nTRT	sTRT	*p*
CIT (h)	14.7 (SD: 4.3)	14.4 (SD: 4.8)	n.s.	14.9	12.8	n.s.
HT (min.)	42 (SD: 17)	35 (SD: 13)	*0.02*	38	36	n.s.
OT (min.)	132 (SD: 40)	152 (SD: 37)	*0.02*	118	142	n.s.
Stent use %	13.3	25.0	n.s.	11.1	11.1	n.s.
DGF %	22.4	60.0	*0.00009*	22.2	44.4	n.s.
Haematoma %	16.6	35.0	*0.031*	22.2	55.6	n.s.
Lymphocele %	4.0	0	n.s.	11.1	0	n.s.
Vascular complications %	2.94	5.0	n.s.	0	11.1	n.s.
Ureter complications %	8.7	20.0	n.s (0.08)	11.1	33.3	n.s.
Reoperations within 30 days, %	8.5	10.0	n.s.	11.1	22.2	n.s.
Biopsy %	22.4	40.0	n.s. (0.06)	33.3	66.7	n.s.
Acute rejection %	15.9	15.0	n.s.	22.2	22.2	n.s.
Hospital stay (days)	14.8	21.4	*0.003*	11.7	28.8	*0.0213*

Considered signficant if *p* < 0.05

We did not observe differences in vascular or ureter-related complications, and there were no more lymphoceles. In the TRT group, one arterial thrombosis caused graft loss; in the PRT group, we could save three kidneys out of 19 having vascular complications. The follow-up US revealed several perirenal hematomas, but they did not require more reoperation.

There were no differences in acute rejection rate (PRT: 15.9% vs. TRT: 15%).

The DGF rate proved to be much more frequent: 22.4% in the PRT and 60% in the TRT group, which is highly significant. In case of TRT, the OR of DGF is 5.2 (2.1–12.9).

Hospital stay proved to be roughly a week longer (PRT 14.8 vs. TRT 21.4 days); propensity analysis verified the significance.

Out of PRT, 78.2% got end-to-side uretero-ureteral anastomosis, while in the TRT group, 70% of recipients received neocystostomy; this difference is highly significant (see Table 4).

**Table 4:** Types of ureter anastomosis

	ureteric	Neocystostomy	*p*
PRT	78.2% (504)	21.7% (140)	*0.00*
TRT	30% (6)	70% (14)	

Considered significant if *p* < 0.05

### Patient survival

**Fig. 1:**
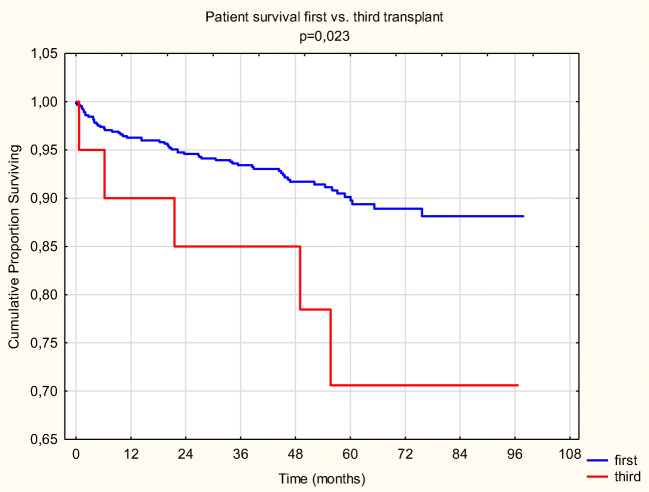
Patient survival PRT vs. TRT

Patient survival at 1, 3, and 5 years for PRT is 96.4%, 93.9%, and 91.2%, and for TRT it is 90%, 85%, and 78.4%, respectively (*p* = 0.023) (Fig. 1). Propensity score matching analysis reconfirmed the significance of the difference.

In the first 30 days, there were 3 (0.46%) deaths in the PRT and 1 (5%) in the TRT group (*p* = 0.0014). Until March 2019, we lost 56 more patients in the PRT group (∑: 59, which is 9.13%) and 4 more in the TRT group (∑: 5, which is 25%), resulting in a significant difference (*p* = 0.01).

The OR of a fatal outcome for TRT patient is 3.3 (1.16–9.4) compared to PRT.

Patient survival at 1, 3, and 5 years for nTRT is 100% and for sTRT is 77.7%, 66.7%, and 66.7% (*p* = 0.065).

### Graft survival

**Fig. 2:**
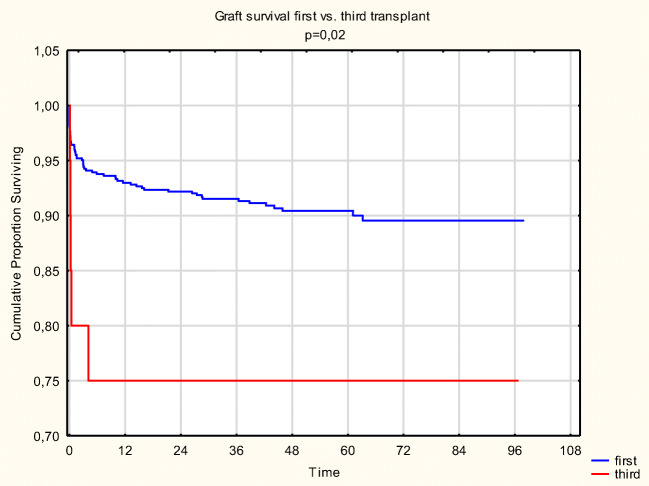
Graft survival PRT vs. TRT

Death censored graft survival at 1, 3, and 5 years for PRT is 93.1%, 91.4%, and 90.3%, and for TRT, it is 75%, 75%, and 75%, respectively (*p* = 0.020) (Fig. 2). Propensity analysis revealed better graft survival for PRT; however, this was not significant.

In the first 30 days, there were 26 (4.02%) graft losses in the PRT and 4 (20%) in the TRT group (*p* = 0.00069). One graft had arterial thrombosis caused by acute accelerated rejection; one had acute irreversible rejection; in the others, ischaemic lesions were observed even though ultrasound showed proper circulation.

We lost one more graft about 4 months later due to acute bacterial nephritis following several reoperations. In the long run, there were no more TRT graft losses.

From 2011 to March 2019, graft loss in surviving patients was 9.6% in the PRT and 25% in the TRT group (*p* = 0.024).

The odds ratio of graft loss for TRT recipients is 3.14 (1.1–8.9) compared to PRT.

Graft survival at 1, 3, and 5 years for nTRT is 100% and for sTRT is 55.6% (*p* = 0.028).

Patient and graft losses are summarized in Table 5.

**Table 5:** Graft and patient loss

	All recipients			Paired recipients		
	PRT	TRT	*p*	nTRT	sTRT	*p*
Graft loss within 30 days %	4.02	20	*0.00069*	0	33.3	n.s. (0.058)
Graftectomy within 30 days, %	3.25	5.0	n.s.	0	11.1	n.s.
Graft loss total, %	9.6	25	*0.02411*	0	44.4	*0.02334*
Death within 30 days, %	0.46	5.0	*0.0014*	0	11.1	n.s.
Death total, %	9.13	25	*0.01*	0	33.3	n.s. (0.058)
Efficiency (working grafts) %	85.76	60.0	*0.00149*	100	44.4	*0.00851*

Considered significant if *p* < 0.05

### Kidney function

In the TRT group, the function of surviving kidneys is moderately and continuously inferior (see Table 6).

**Table 6:** Postoperative serum creatinine (μmol/l)

	PRT	TRT	*p*
Preop	659	738	0,096
Day 1	544	665	0,002
Day 3	397	597	0,001
Day 5	330	453	0,016
Day 7	280	380	0,065
Day 10	223	316	0,076
Month 1	154	219	0,107
Year 1	140	138	0,631

**Fig. 3:**
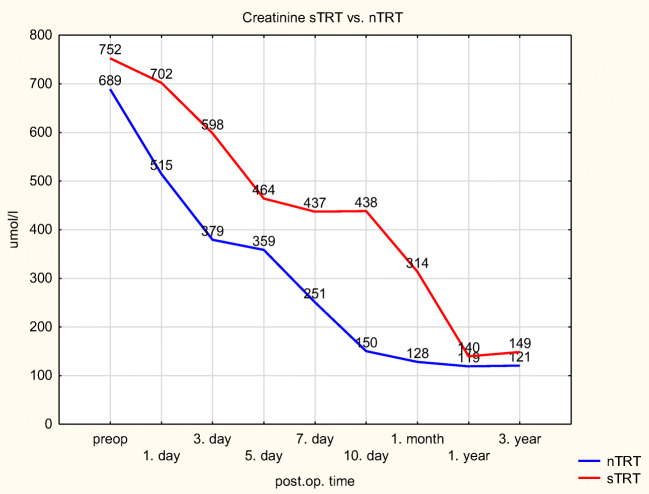
Kidney function nTRT vs. sTRT

Comparing the sTRT and nTRT groups, who share the same donor, this difference is present, but it does not reach statistical significance either (see Fig. 3).

## Discussion

### Donors

We did not perform any TRT from an ECD. We acknowledge that a selection bias is likely to exist at that point, favouring the TRT group. This practice is justified by the observation of Miles et al.: The survival of ECD retransplant recipients was not different from those remaining on dialysis [19]. The quality of kidneys used for TRT is the same or even better than the quality of those used for PRT [3, 16].

### Recipients

Our rate of TRT, 2.6%, belongs into the upper range according to published data, varying from 1–1.4% [16, 20] to 2.4–2.9% [10, 14].

There were no really obese patients in our TRT group. A selection bias might exist at that point, too; a real obese candidate (BMI > 35) would be refused, as obesity poses a significant risk of surgical complications [21–23].

Many centres have observed that TRT patients are younger [2, 3, 14, 16, 20]. Third recipients at a younger age already have a long, chronic medical history. These repeated Uremia-dialysis-immunosuppression-surgery sequences obviously alter the patient’s body structure, cardiac and vascular status, etc. [11, 16, 24, 25]. This factor is probably highly underestimated as it is almost impossible to objectify. Kousoulas et al. presented a cut value, 43 years, as an independent risk factor for mortality [26]. Further efforts are to be taken to prevent early graft loss in these young patients with long predicted lifespan.

The rate of HCV positivity in the PRT group is slightly higher than the population-based frequency in Hungary (0.5–0.7%) [27, 28]. The infection rate reaches a tenfold higher rate in the TRT group, resulting in a highly significant difference.

The PRA level, a marker of prior sensitization, proved to be much higher in the TRT population. This is expected and consistent with all the known literature. Several papers report a trend that a PRA of more than 80% is associated with poor long-term graft function [15, 20, 29]. In our cohort, we could not demonstrate a significant relationship between the PRA level and the outcome. Out of twenty, only two of our TRT recipients had PRA > 80%, and both are doing well. This remains a controversial area of transplantation.

### Surgery, postop course, rejection

The mean cold ischaemic time is the same for PRT and TRT. Comparing the nTRT and sTRT groups, there is an almost 2-h difference in favour of third transplantations.

TRT needs significantly longer operative time [3, 26, 30].

Surgical opinions vary in the question of previous transplant nephrectomy. Kienzl-Wagner et al. claim it is not necessary at all [25]. Another opinion is that it can be performed in the same setting prior to retransplantation [15, 24, 31].

Our policy is that at least one of the previous grafts has to be removed prior to waitlisting. We strongly believe that this is rational as both presence and removal might cause unexpected complications. TRT itself is a demanding long operation performed on a comorbid patient, and there is no need for any extension of the procedure.

Another potential surgical challenge is creating ureter anastomosis. The majority of centres perform neocystostomy for TRT patients [20, 25, 32], and uretero-ureteric anastomosis is reserved for technical difficulties [33]. Historically, a specialty of our centre is the creation of end-to-side uretero-ureteral anastomosis [34, 35]. But in the third transplant procedures, the native ureter of the recipient is more likely to be scaring or tight, and because of that, in most of the cases, neocystostomy has been performed. Yet, more ureter complications occurred in TRT than in PRT, not being significant, but seeming to be remarkable clinically.

Induction therapy was given after immunologic consideration in a few cases in PRT and to everyone in the TRT group with the likely result that there was no difference in acute rejection rates. This is important as the negative impact of even a single episode of acute rejection is well documented [20, 36–39]. However, this finding is in contrast with others [3, 12, 20].

The DGF rate proved to be significantly higher in TRT. Most authors assume that the increased rate of DGF is driven by recipient factors, namely, sensitization, rather than by donor factors [3, 16, 20, 25, 30, 40, 41].

Remarkably more indicative biopsies were obtained in TRT. We do not perform protocol biopsies.

Induction therapy, prolonged DGF, more frequent biopsy, and other factors resulted to a 1-week longer hospital stay, reaching a high significance.

All these factors together count for much higher expenses in case of a third transplantation [42, 43].

### Outcome

#### Patient survival

Death in the first 30 days occurred in very few cases, due to cardiac failure or pneumonia with sepsis and ARDS. On the long run, cerebro- and cardiovascular events caused the death of our TRT patients, who are at a higher risk of fatal outcome. Our finding corresponds to the multicentre ET study [14]. Many other groups reported similar patient survival as compared to first and second transplants, but the leading causes of death, cardiovascular events and sepsis, correlate with our findings [3, 10, 12, 13, 24, 25, 44].

On the other hand, even third transplantation is associated with a significant survival advantage relative to remaining on dialysis, provided that an SCD donor organ was used [4, 6, 11, 16, 19].

#### Graft survival

Graft losses occurred in the first 6 months, and in our paired subpopulation (sTRT vs. nTRT), the same was observed. This early graft loss is likely the cause of inferior graft survival [2, 16, 25, 30, 31]. Assfalg et al. in the ET study observed significantly worse patient and graft survival, and the authors found this issue so pronounced that they question the current policy of repeated retransplantations, especially the forth ones [14]. However, Horovitz et al., who compared the kidneys from paired donors, demonstrated only insignificant differences [3].

We had no TRT graft loss due to surgical reasons. Graft loss occurred as the result of either early rejection or chronic allograft dysfunction, which is in agreement with others [3, 10, 20, 25, 30].

We introduced a not too scientific, but practical parameter to assess the efficiency of our labour investment into third transplantations. The rate of working kidneys in living recipients per performed transplantations is much higher following PRT.

TRT and sTRT recipients show a lower GFR until the end of the first post-transplant year. This might have a clinical importance, although mathematically it is not significant.

## Summary

In a third transplant, younger recipients receive a younger, good quality kidney, and still both graft and patient survival are significantly inferior to primer ones. Third kidney transplantations may be performed safely by experienced, senior surgeons, but they represent an intense surgical challenge. The main cause of graft loss is rather immune mediated than surgical. This sensitized patient population requires profound immunosuppression with all its risks and consequences.

Patients are at a high mortality risk receiving a third transplant, but this is probably less than remaining on dialysis. A meticulous patient selection is mandatory with a view to reducing post-transplant mortality. Immunotherapy, postoperative dialysis, and prolonged hospital stay cause remarkable expenses. Further prospective studies should be performed to compare the third transplantation with the continuation of dialysis.

## Data Availability

Data are available at the corresponding author.

## Abbreviations

ARDS: Acute respiratory distress syndrome
ATG: Anti-thymocyte globulin
BMI: Body mass index
CIT: Cold ischaemic time
CMV: Cytomegalovirus
CT: Computed tomography
DBD: Donation after brain death
DCD: Donation after cardiac death
DGF: Delayed graft function
ECD: Expanded criteria donor
ET: Eurotransplant
HCV: Hepatitis C
HLA: Human leukocyte antigen
HT: Handling, or “anastomosis” time
KDPI: Kidney donor profile index
KDRI: Kidney donor risk index
MA: Mycophenolic acid
nTRT: Non-third renal transplant
OR: Odds ratio
OT: Operation time
PRA: Panel-reactive antibodies
PRT: Primer renal transplant
SCD: Standard criteria donor
SD: Standard deviation
sTRT: Selected third renal transplant
TAC: Tacrolimus
TRT: Third renal transplant
US: Ultrasound
